# Exploring the potential of electrical bioimpedance technique for analyzing physical activity

**DOI:** 10.3389/fphys.2024.1515431

**Published:** 2024-12-19

**Authors:** Abdelakram Hafid, Samaneh Zolfaghari, Annica Kristoffersson, Mia Folke

**Affiliations:** Division of Intelligent Future Technologies, School of Innovation, Design and Technology, Mälardalen University, Västerås, Sweden

**Keywords:** electrical bioimpedance, muscle activity, physical activities, human motion recognition, signal characterization, lower body movement

## Abstract

**Introduction:**

Exercise physiology investigates the complex and multifaceted human body responses to physical activity (PA). The integration of electrical bioimpedance (EBI) has emerged as a valuable tool for deepening our understanding of muscle activity during exercise.

**Method:**

In this study, we investigate the potential of using the EBI technique for human motion recognition. We analyze EBI signals from the quadriceps muscle and extensor digitorum longus muscle acquired when healthy participants in the range 20–30 years of age performed four lower body PAs, namely squats, lunges, balance walk, and short jumps.

**Results:**

The characteristics of EBI signals are promising for analyzing PAs. Each evaluated PA exhibited unique EBI signal characteristics.

**Discussion:**

The variability in how PAs are executed leads to variations in the EBI signal characteristics, which, in turn, can provide insights into individual differences in how a person executes a specific PA.

## 1 Introduction

Exercise physiology, which is a multidisciplinary domain, explores the sophisticated mechanisms that dictate the human body’s physiological response to exercise. This field serves as a nexus between biomechanics, physiology, and biochemistry (Kent and Hayes, 2021), shedding light on the intricate dynamics among these systems and their respective adaptations in response to exercise. Physical activity (PA), a fundamental aspect of human existence, is defined as any energy-consuming body movement produced by skeletal muscles and includes a broad array of movements ranging from everyday tasks to intentional exercises such as walking, running, martial arts, and more (Posadzki et al., 2020). Over the decade spanning from 2012 to 2022, there was a significant surge in research publications on human motion wearables, with an annual count escalating from roughly 250 to nearly 1,400 articles (Huang et al., 2023). There is an interest in measuring a person’s PA both for the individual, the healthcare services, and for different research fields. There is also an interest in determining what movement is performed and how it is executed.

Various techniques are employed for human motion recognition, PA monitoring, and posture assessment. Three-dimensional Optical Motion Capture systems are considered the golden standard method for motion capture (Hindle et al., 2021; Gu et al., 2023). Renowned for their provision of highly accurate and comprehensive motion data, they play a pivotal role in these domains. However, their utility is constrained by the necessity for a controlled environment, susceptibility to occlusion, and the relatively higher complexity and computational cost (Gutemberg, 2005) compared to Inertial Measurement Unit (IMU)-based techniques. IMUs, on the other hand, have found extensive application across diverse fields (Huang et al., 2023; Meng et al., 2020), particularly in sports, where they are utilized for the classification of human motion through various classification approaches (Kranzinger et al., 2023). Despite offering notable advantages in terms of ease of use, flexibility, and cost-effectiveness, IMU-based techniques necessitate careful consideration of sensor drift (Cheng et al., 2023), sensor placement (Niswander et al., 2020), and environmental factors (Patrizi et al., 2022) to ensure the accuracy and reliability of human motion recognition and monitoring.

Three-dimensional Optical Motion Capture systems or IMU sensors measure the direction of the body movement but do not analyze the muscles involved. IMU sensors have shown limited capability to detect age related deteriorations in how to perform a PA (Swanson and Fling, 2021). Over the past decades, electrical bioimpedance (EBI) has emerged as a versatile and non-invasive tool, offering valuable insights into diverse aspects of physiological function (Naranjo-Hernández et al., 2019; Platt, 1998). In fields such as sports and exercise research and practice, novel approaches for measurements utilizing EBI are continuously evolving. By assessing the resistance of biological tissue to electrical current flow, EBI encompasses several modalities, such as bioelectrical impedance analysis (BIA), a method employed for assessing body composition and recognized for its non-invasive nature. It is also utilized to evaluate hydration and injury, particularly yielding optimal results when applied to soft tissue injuries (Castizo-Olier et al., 2018). The recent strides in microelectronics have catalyzed the creation of system-on-chip solutions tailored specifically for EBI. These advancements have enhanced the portability and user-friendliness of EBI devices significantly, rendering them highly suitable for both research endeavors and practical applications.

Electrical impedance myography (EIMG), among the modalities of EBI, is used in clinical settings and research domains for assessing muscle conditions. It operates by measuring the resistance in biological tissues, a parameter influenced by both tissue geometry and composition (Cebrián-Ponce et al., 2021). EIMG can be used for the detection of inflammation in muscles (Mortreux et al., 2019) and muscle fatigue (Huang et al., 2020), and in evaluation of neuromuscular disorders (Sanchez and Rutkove, 2017).

EIMG has also been evaluated for measurements of muscle status before and directly after exercise, the week after exercise, and a longer time after exercise (Cebrián-Ponce et al., 2021). Changes in impedance magnitude and phase angle were shown to decrease after exercise compared to before exercise (Huang et al., 2020; Freeborn and Fu, 2019; Fu and Freeborn, 2018). Measurements performed during muscle contraction show a nonlinear increase in the resistance and reactance (Shiffman et al., 2003). The phase angle is directly related to the strength of the muscle and aerobic fitness (Martins et al., 2022). However, even though there are several studies investigating the topic, EIMG is still a new method for muscle assessment and the technology is far from being considered as a consolidated technique (Sanchez et al., 2021). The underlying mechanisms involved in EIMG during muscle contraction are not fully known (Sanchez et al., 2021).

By using EBI technology for human motion recognition, it might be possible to get information on how the muscles handle PAs performed. In the current literature, limited studies focus on human motion recognition utilizing EBI. From 2015 to 2023, investigations have successfully utilized EBI specifically used different electrode positions and algorithms for hand gesture recognition (Zhang and Harrison, 2015; Chen et al., 2021; Ma et al., 2023). A notable study published in January 2024 illustrates the utilization of single-channel EBI sensing between wrists, and IMUs, for recognizing various upper and lower body PAs (Liu et al., 2024). To the best of our knowledge, there are no other studies focusing on human motion recognition utilizing EBI.

In this study, we aim to investigate the potential of using the EBI technique for human motion recognition and thereby be able to analyze more than the change in body directions that occur while performing different PAs. By analyzing EBI signals from the quadriceps muscle and the extensor digitorum longus muscle acquired during PAs, the study seeks to uncover the characteristics of the EBI signals for four different lower body PAs (squats, lunges, balance walk, and short jumps) for healthy young people.

## 2 Materials and method

This section first describes the experimental measurement setup and the data collection procedure. Secondly, it describes the data processing and characterization of the EBI signals, as well as the feature extraction and calculation.

### 2.1 Experimental measurement setup

To enable the investigation of the potential of using EBI measurements on specific muscles as a technique for human motion recognition, we employed two EBI devices, the Max30009EVKIT^1^ and Max86178EVKIT^2^. Each kit integrates a system-on-chip functionality, serving as a low-power, high-performance analog frontend for EBI measurements. Both kits include a microcontroller board and are powered by rechargeable LiPo batteries. Moreover, both can establish communication with dedicated graphical user interface (GUI) software via Bluetooth.

The rationale behind selecting and using two different types of EBI devices was due to encountering a problem where two identical kits could not operate simultaneously on the same computer due to GUI limitations and Bluetooth connection issues with identical devices.

The Max30009EVKIT is tailored for EBI measurements and serves a wide range of applications from wearable fitness devices to medical equipment. Conversely, the Max86178EVKIT not only provides EBI measurement capabilities but also includes features for measuring photoplethysmography (PPG) signals and electrocardiography (ECG) readings. Its versatility extends to smart clothing applications and the development of wearable vital sign monitors.

The sensor locations were influenced by the muscles’ capacity to assess the engagement of lower body muscles during various PAs. The quadriceps muscle was chosen since it is a large muscle involved in leg extension, and the extensor digitorum longus muscle was chosen based on its involvement with flexion of the ankle and foot stabilization. The extensor digitorum longus muscle is important to compensate for imbalance.

**FIGURE 1:**
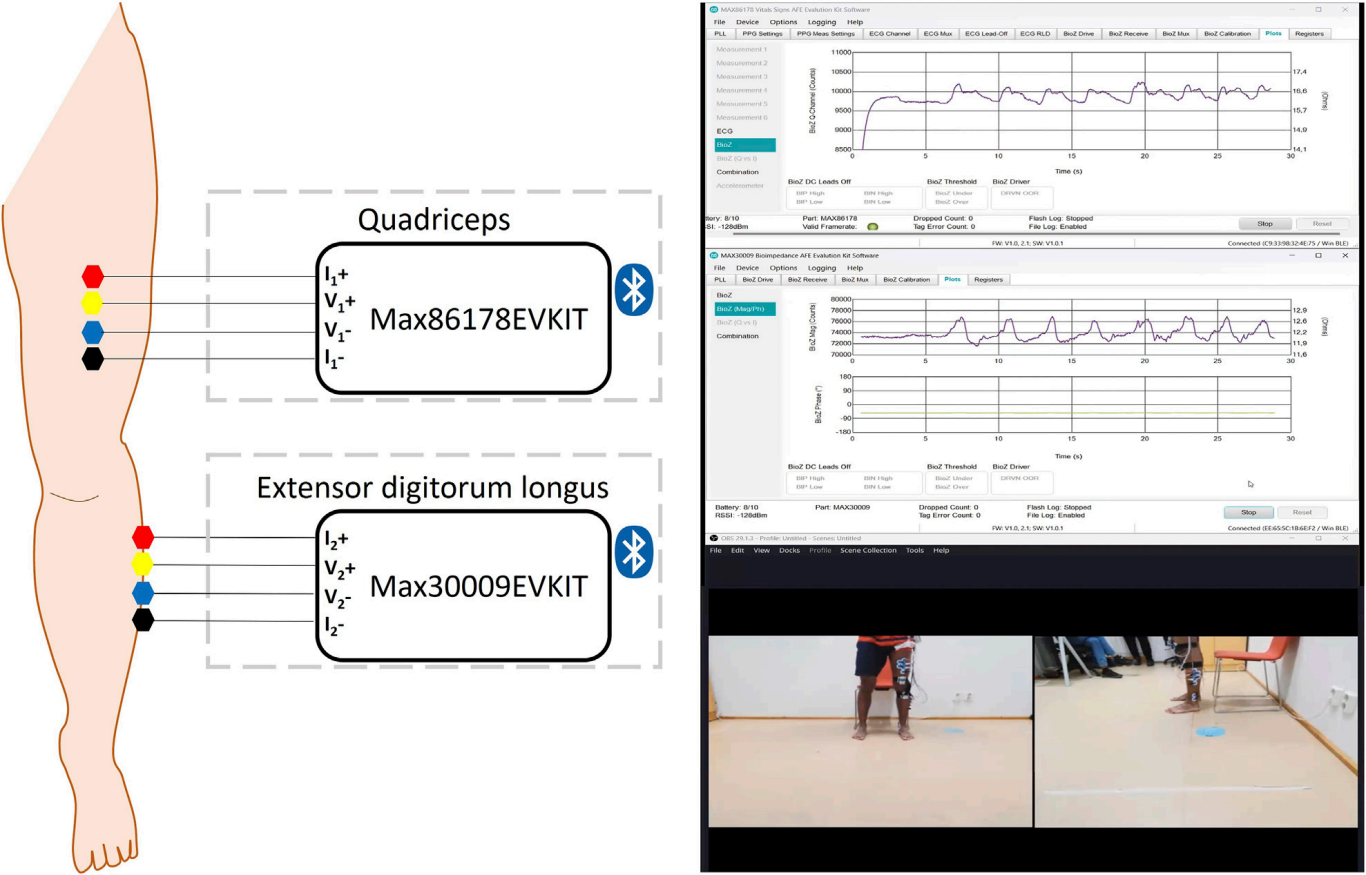
Arrangement of Ag/AgCl gel electrodes with four electrodes on the quadriceps muscle and an additional four on the extensor digitorum longus muscle. A screenshot of the Max30009EVKIT and Max86178EVKIT GUIs and the recorded video.

As shown in Figure 1, a total of eight Ag/AgCl gel electrodes were positioned on one leg to facilitate the EBI measurements. Based on a tetrapolar configuration, four electrodes were placed on the quadriceps muscle and an additional four electrodes on the extensor digitorum longus muscle. In this setup, the voltage-sensing electrodes were assigned to the inner electrodes labeled as V, while the outer electrodes, labeled as I, functioned as the current-injecting electrodes.

The electrode configuration was selected to minimize the influence of the skin-electrode interface on the measurement (Kalvøy et al., 2018). In addition, hair was shaved to reduce baseline artifacts. The Max86178EVKIT was utilized to collect data from the quadriceps muscle, while the Max30009EVKIT was employed for data acquisition from the extensor digitorum longus muscle. Both kits were carried in a bag fastened around the participants’ waist.

In this study, the injected current was an alternating current set at frequencies of 50 kHz, with an amplitude of 1.28 mA for both devices. The EBI signals for both devices were captured at a maximum sampling rate of 390 Hz.

Additionally, two cameras (UltraSharp Webcam - WB7022) were utilized to capture the participants’ PAs during the entire measurement process. To preserve anonymity and maximize video quality, the camera angles were carefully selected to focus solely on the lower part of the body where the electrodes were positioned, with recordings taken from both lateral and frontal perspectives. OBS Studio (29.1.3) software was employed to display and record the feeds from the two cameras simultaneously, as depicted in Figure 1.

### 2.2 Data collection procedure

The experimental evaluation adhered to the ethical guidelines sanctioned by the Swedish Ethical Review Authority under the reference code 2022-06690-01. All procedures were conducted in strict accordance with the principles outlined in the Declaration of Helsinki.

The experimental evaluation, which took place as part of the PRE-fall project^3^, involved 11 young healthy volunteers (8 males and 3 females, aged 20–30). Before placing electrodes and commencing measurements, all participants received detailed information regarding the study, and each participant provided informed consent.

Since the measurements were performed both to investigate the characteristics of the sensor data and if imbalance occur during the PAs, we collected EBI data from the participants’ non-dominant leg. We therefore asked each participant to stand on one leg to determine the dominant leg. The electrodes were placed on the other leg, from here on referred to as *the sensor leg*.

**FIGURE 2:**
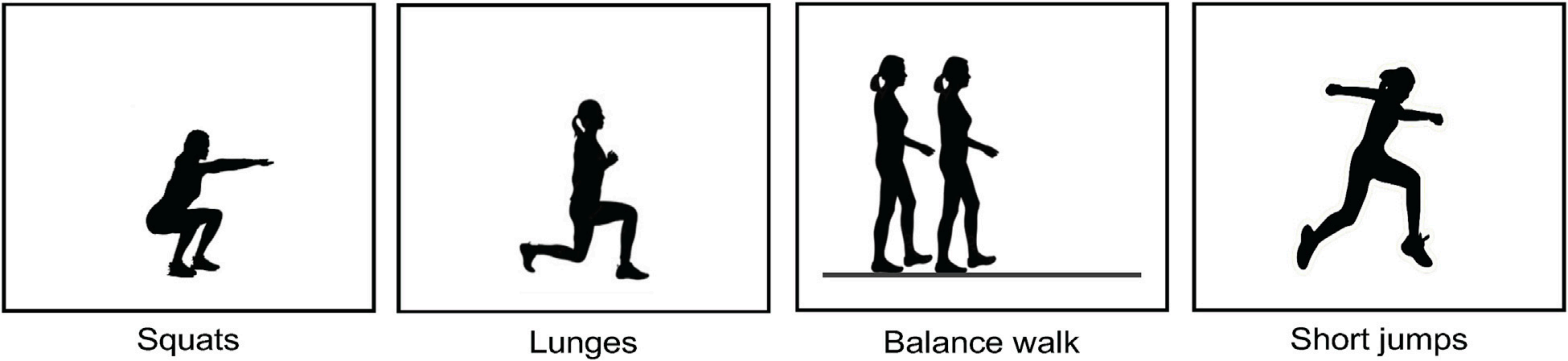
The PAs performed in the experimental evaluation.

The participants performed a series of four PAs (squats, lunges, balance walk, and short jumps) as depicted in Figure 2. The choice of these PAs was driven by their substantial relevance to functional movements and their ability to engage multiple muscles in the lower body. The participants were orally and visually instructed on how to perform the PAs described below:

• For the squats, the participants were told to perform 5 squats as fast as possible. Participants should bend their knees and descend quickly toward a chair, without sitting down, and then stand up again as quickly as possible. The chair guided squat depth.
• For the lunges, the participants were told to perform 3 lunges. Participants should take a large step forward with the sensor leg, bend their legs to approximately 90°, and then return to the starting position without making ground contact with the sensor leg until fully returned to the starting position.
• For the balance walk, the participants were told to walk in a straight line with shortened strides, ensuring that the heel of the front foot was close to the toe of the trailing foot.
• For the short jumps, the participants were told to perform a 70 cm jump with the sensor leg in front of the other one, followed by walking backward to the starting position for making a subsequent jump until 3 jumps were performed.

To facilitate the data analysis, the participants were told when to start performing each PA. A shorter resting period was provided between the successive PAs.

### 2.3 Data processing and characterization of EBI signals

The data recorded from the Max30009EVKIT and Max86178EVKIT GUI software was stored in two raw datasets.

The recorded videos for each participant and the corresponding EBI sensor data for the four PAs were carefully analyzed using the ELAN annotation tool (Wittenburg et al., 2006). Two of the authors reviewed each video together to determine the start and end times for the PAs. Each video sequence was viewed in slow motion (video speed 0.5) at least three times to assess the impact of each directional change of the PA on the two EBI signals and to identify any observable similarities and differences in EBI signals obtained from different participants. During this process, casual labels indicating PA cycles were assigned to the two EBI signals. Additionally, how the participants executed the PA was analyzed by reviewing the videos.

EBI signals that did not display the typical characteristics observed in other participants’ EBI signals were excluded from the analysis. The number of included EBI signals for each PA and muscle, and information on which EBI signals that were excluded are detailed in Table 1.

**TABLE 1:** Included and excluded EBI signals for each PA and muscle.

PA	Muscle	No of included participants	Excluded participant id:s
Squats	Quadriceps muscle	10	5
	Extensor digitorum longus muscle	11	—
Lunges	Quadriceps muscle	11	—
	Extensor digitorum longus muscle	8	4,5,8
Balance walk	Quadriceps muscle	9	6,7
	Extensor digitorum longus muscle	8	6,7,11
Short jumps	Quadriceps muscle	7	1,3,4,10
	Extensor digitorum longus muscle	9	10,11

For the included EBI signals, Daubechies wavelet filtering using the “db4” wavelet from the “pywt” library in Python version 3.10.13 was applied to the raw EBI signals to suppress noise and retain critical signal components. This filtering method involves decomposing the EBI signal into wavelet coefficients through a multi-level discrete wavelet transform, which isolates noise from the signal across different scales. Soft thresholding was then applied to the wavelet coefficients, reducing those below a threshold of 0.1. The threshold was determined experimentally. The signal was subsequently reconstructed from the thresholded coefficients using an inverse wavelet transform. To maintain consistency with the raw signal, the reconstructed signal was truncated to match the original length. The final output was the processed EBI signal.

To show the characteristics of the EBI signals and variations in them, figures representing the signal characteristics of the processed EBI signals obtained from the PAs were plotted using the “matplotlib”^4^ library. Phases within each cycle were highlighted in the figures. These phases were identified by analyzing the EBI signals and the corresponding videos simultaneously. For squats and lunges, the EBI signal from the participant who executed the PA most effectively while providing the clearest EBI signals were provided as examples to show the phases in the PA. For balance walk and short jumps, the phases were presented in all figures since the EBI signal characteristics are less distinct and sometimes, the phases were impossible to detect without video observations. The number of participants displaying different variations in the EBI signal characteristics for each PA was also counted.

### 2.4 Feature extraction and calculations

The “SciPy” scientific computation library^5^ was used to detect and extract the squat/lunge cycles from the EBI signals of the included participants. The proposed algorithm analyzes the EBI signals collected from each of the two muscles by identifying prominent peaks and local minima within the EBI signals. The pseudo-code of the cycle extraction algorithm is provided in Algorithm 1.

Algorithm 1. Cycle Extraction (*EBI signal:* A one-dimensional array representing the signal data).

1: Establish a threshold for prominent peaks. This is determined by calculating the difference between the absolute maximum and the mean value of the entire EBI signal.
2: Detect the prominent peaks. This is done by selecting the most prominent peaks exceeding the threshold from the entire EBI signal. The minimum distance between the prominent peaks was experimentally determined to 640 samples, a value corresponding to almost the length of a full cycle.
3: Determine the *‘Start cycle index’* and *‘End cycle index’* for each cycle:
  **for** squats, the *‘Start cycle index’* and *‘End cycle index’* correspond to the first prominent local minima prior to, and after, a prominent peak.
  **for** lunges, the *‘Start cycle index’* and *‘End cycle index’* correspond to the second prominent local minima prior to, and after, a prominent peak.
***return*** *cycles:* A list representing the start and end indices of each identified cycle in the EBI signal.

It should be mentioned that, since the squats were performed at maximum speed, the first and last of the five squats cycles were excluded from further analysis to ensure accuracy. As a result, three cycles were selected for feature extraction and analysis from each included participant. However, for P1, squat cycles 1, 2, and 5 were included in the analysis due to balance issues impacting squat cycles 3 and 4.

For lunges, all three cycles were used in the feature extraction and analysis from each included participant.

**FIGURE 3:**
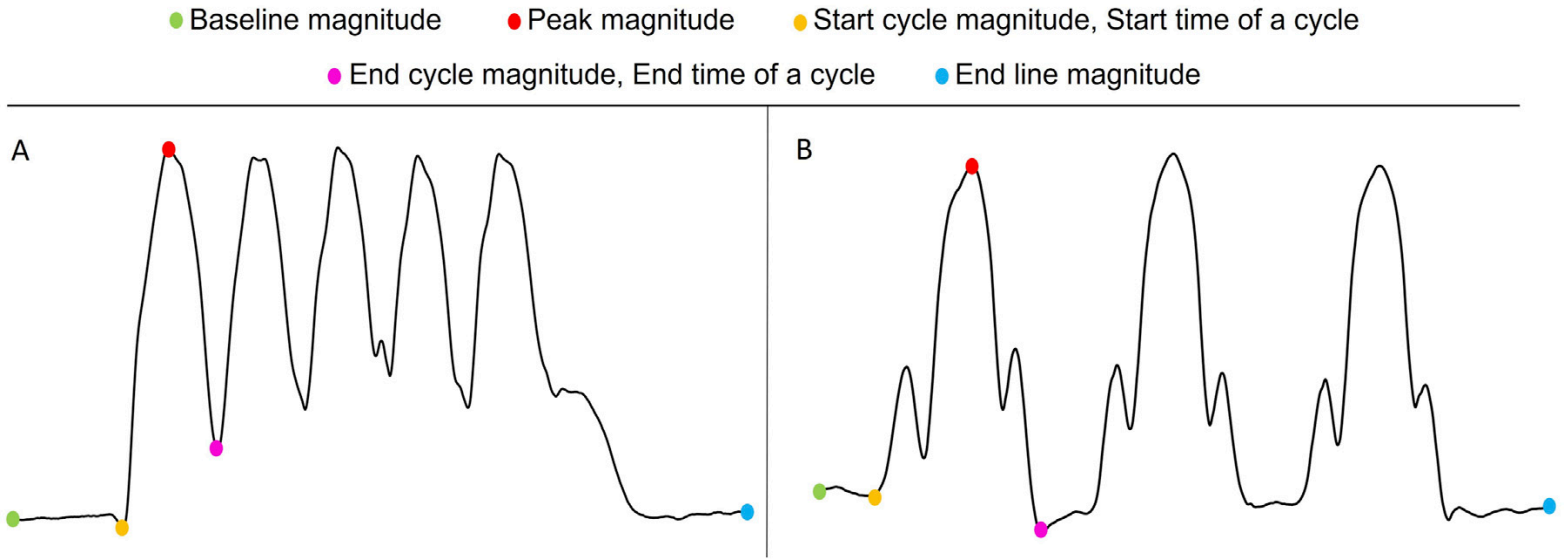
Visualization of measurable features in an EBI signal collected during squats **(A)** and lunges **(B)**.

For both squats and lunges, the following measurable features, as depicted in Figure 3, were extracted:

**Baseline magnitude:** The impedance value before the start of all cycles (participant in standing position). It reflects the impedance when the muscle is relatively relaxed.
**Endline magnitude:** The impedance value after all cycles (participant in standing position). It reflects the impedance when the muscle is relatively relaxed.
**Start cycle magnitude:** The impedance value at the start time of a cycle.
**End cycle magnitude:** The impedance value at the end time of a cycle.
**Peak magnitude:** The highest impedance value corresponding to the squat/lunge position in each cycle.

In addition, the following features were calculated for each squat/lunge cycle:

**Squat/Lunge position amplitude:** $Peak\;magnitude-\frac{Start\;cycle\;magnitude+End\;cycle\;magnitude}{2}$ . The impedance position amplitude is indicative of the degree of muscle contraction and the range of motion during the squat/lunge cycle. A large squat/lunge position amplitude reflects a larger muscle mass involvement.
**Baseline to peak amplitude:** Peak magnitude−Baseline magnitude. The impedance value reflects the muscle mass.
**Number of fluctuations:** The number of occasions where a local minima and local maxima occur in close proximity to each other during a squat/lunge cycle. It reflects the imbalance and coordination problems during each cycle.

The pseudo-code of the algorithm for detecting fluctuations for squat/lunge cycles is provided in Algorithm 2.

Algorithm 2. Fluctuation extraction *(EBI_signal:* A one-dimensional array representing the signal data of one cycle).

1: Identify all peaks (local maxima) and valleys (local minima).
2: Identify the prominent peak, i.e., the peak with the highest amplitude.
3: Identify the two local minima occurring prior to and after the prominent peak.
  **IF** the distance between the prominent peak and either local minimum is less than 5 data points, remove the prominent peak and the two local minima from the list of peaks and valleys.
  **ELSE** remove only the prominent peak from the list but retain its index for step 4.
4: Divide remaining peaks and valleys into a left-side group and right-side group based on those occurring prior to or after the prominent peak.
5: Compare left-side peaks and their corresponding valleys. Ensure the valley occurs after the peak.
6: Compare right-side peaks and their corresponding valleys. Ensure the valley occurs before the peak.
7: Filter valid matches of peak and valley meeting certain distance and amplitude thresholds. Distance threshold: 5–72 samples between peak and valley. Amplitude thresholds:
  **IF** the distance threshold is met, the amplitude between the peak and valley must be at least 0.005 Ω.
  **ELSE IF** the distance threshold is not met, the amplitude between peak and valley must not exceed 0.014 Ω.
  **ELSE** unvalid match.
***return*** *Filtered peaks and valleys:* A list of indices representing peaks and valleys that indicate fluctuations in the cycle.

Since the participants were asked to perform the squats as quickly as possible, also the following feature was calculated for squats:

Duration: End time of a cycle−Start time of a cycle . The duration reflects the ability to descend into a squat position, stand up, and make directional changes.

The PA cycles for balance walk and short jumps were considered difficult to identify in the EBI signal without simultaneous access to the video recordings. Instead, approximate amplitudes for the respective PA were calculated. For balance walk, the maximum and minimum magnitudes in the EBI signal during the first 8–9 steps were determined. For short jumps, the maximum and minimum magnitudes in the entire EBI signal, i.e., all three short jumps and the walking back in between, were determined. The maximum and minimum magnitudes during balance walk and short jumps respectively were used to calculate the approximate amplitudes for each included participant. Thereafter, the mean value of the approximate amplitudes for all included participants were calculated.

For balance walk, also fluctuations were calculated. However, since the cycles were difficult to identify, the fluctuations occurring during the first 8–9 steps were identified using a similar approach as in the fluctuation extraction algorithm but without detecting the prominent peaks. The number of fluctuations was divided by the number of steps taken to determine the number of fluctuations occurring per step.

To visualize the distribution of these calculated features and differences across the two muscles and the PAs, scatter plots representing average data were obtained for squats, lunges, and balance walk. These scatter plots are presented in Section 3.

## 3 Results

This section presents examples of EBI signals captured from the quadriceps muscle and the extensor digitorum longus muscle of the sensor leg during PA execution. These examples include explanations of the typical signal characteristics of the EBI signal for each PA, as well as explanations of some of the variations observed in the EBI signals. Additionally, the calculated features are presented.

### 3.1 Characteristics of the EBI signals obtained during squats

**FIGURE 4:**
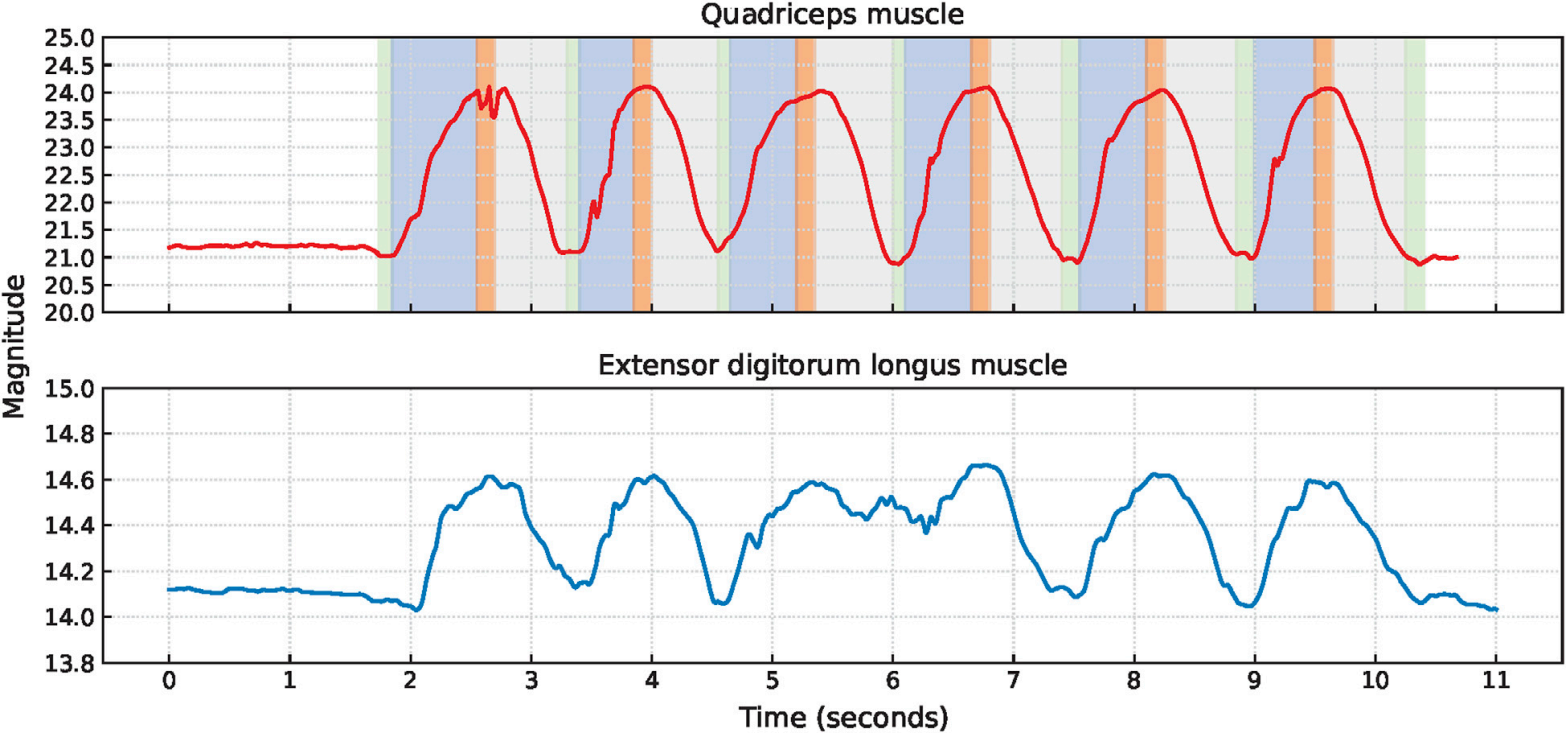
Signal characteristics of the EBI signals for P1 performing squats. The approximate timings for the key phases are color-coded: green for upright position, blue for bending knees, orange for squat position, and gray for stand-up movement in the EBI signal obtained from the quadriceps muscle.

Squat cycles were extracted from the quadriceps muscle for 10 participants and from the extensor digitorum longus muscle for all 11 participants. The signal characteristics of the EBI signals obtained from the quadriceps muscle and the extensor digitorum longus muscle of P1 are presented in Figure 4. The upward slopes represent the bending of the knees, the peaks represent the squat position with knees bent at 90°, the downward slopes represent the stand-up movement, and the valleys represent the upright position. Similar signal characteristics were observed for both muscles, although the EBI signal amplitude was significantly higher for the quadriceps muscle than the extensor digitorum longus muscle. The video showed that the unexpected characteristics in the EBI signal obtained from the extensor digitorum longus muscle at around 6 s in Figure 4, correspond to balance struggles.

#### 3.1.1 Variations in the EBI characteristics

Several variations in the EBI signals obtained during squats were observed, including an elevated baseline (start cycle magnitude higher than baseline magnitude), signal plateaus, and fluctuating signals.

**FIGURE 5:**
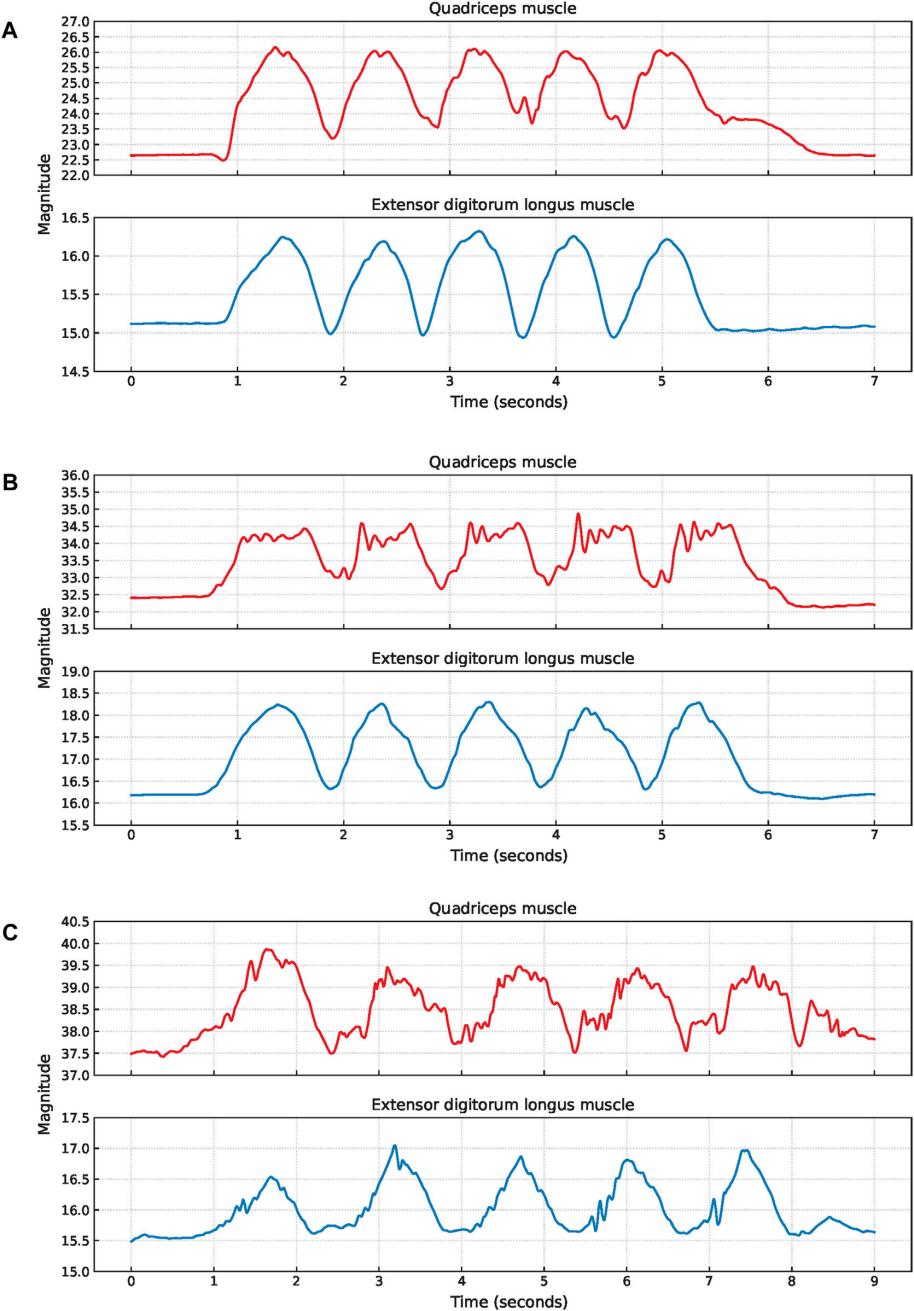
Examples of EBI signal characteristics during squats where **(A)** shows an elevated baseline for the quadriceps muscle of P6, **(B)** shows fluctuating plateaus for the quadriceps muscle of P9, and **(C)** shows fluctuating signals for both muscles of P11.

An elevated baseline was observed in the obtained EBI signal from the quadriceps muscle of six participants. An example of this is shown for P6 in Figure 5A. For three of these observations, the elevated baseline was due to not fully straightening their legs when standing up between the squats. One participant also paused with slightly bent legs after the last squat and straightened them shortly afterward, causing the elevated baseline to return to normal. A similar delay in baseline return to normal after the last squat was also observed for another participant, despite straightening the legs directly after all squats.

Six participants exhibited a plateau in their EBI signals while in the squat position. For three of these participants, this plateau was observed in the EBI signals obtained from both muscles. For two participants, the plateaus were only observed in the EBI signal obtained from the quadriceps muscle. For the remaining participant, it was only observed in the EBI signal obtained from the extensor digitorum longus muscle. Figure 5B provides an example where fluctuating plateaus are observed only in the EBI signal obtained from the quadriceps muscle of P9. The reasons for these plateaus were not visually discernible from the recorded videos.

An example of a fluctuating EBI signal is shown in Figure 5C. The cause of these fluctuations P11 could not be determined based on the video observations.

#### 3.1.2 Calculated features

**FIGURE 6:**
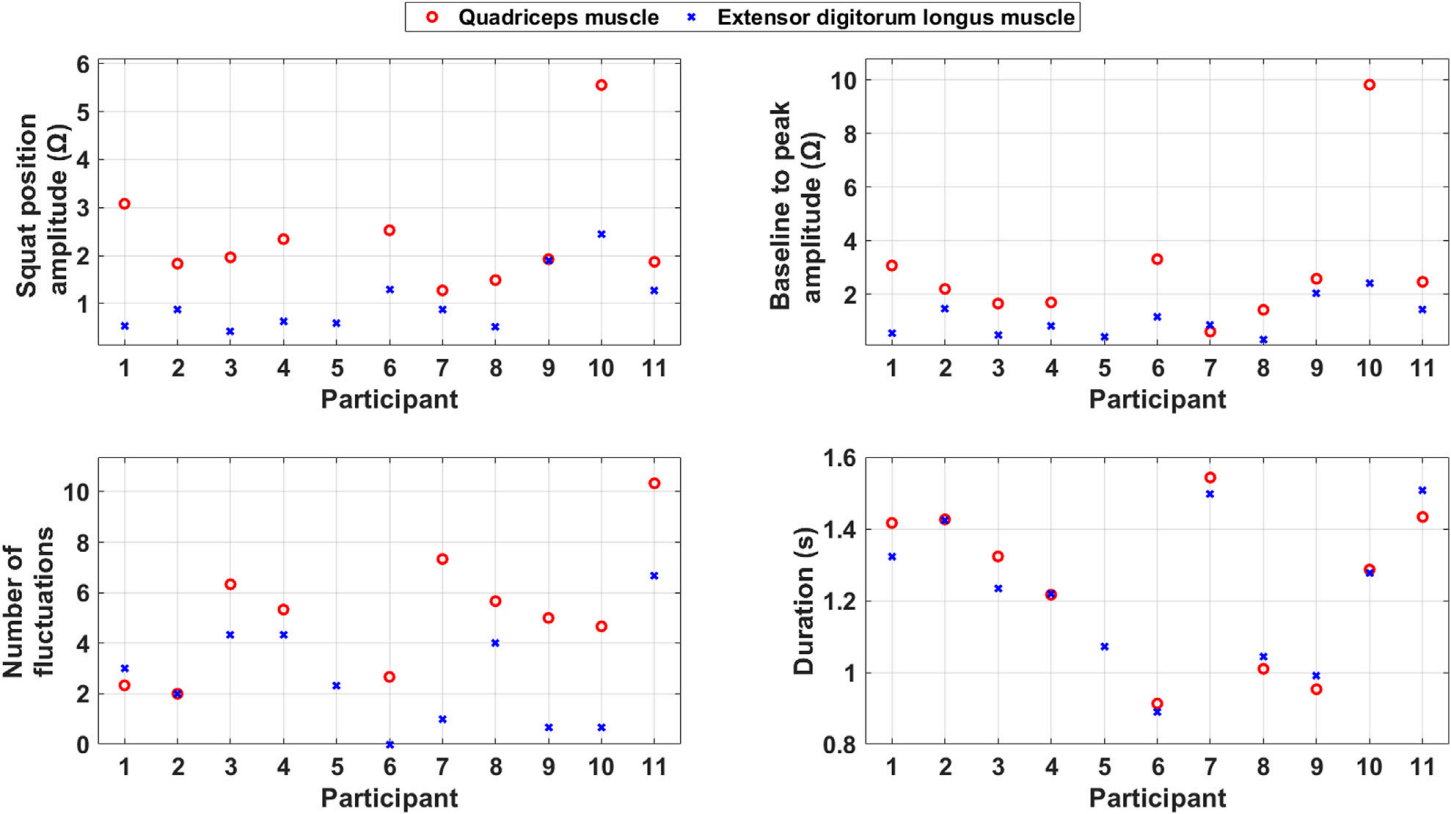
Scatter plots of the average calculated features during squats for each included participant.

Figure 6 shows scatter plots of the average calculated features for each included participant and indicates variability in functionality between the quadriceps muscle and extensor digitorum longus muscles.

The squat position amplitude is generally larger for the quadriceps muscle than the extensor digitorum longus muscle. This indicates that the quadriceps muscle has a stronger and more consistent contraction during the PA than the extensor digitorum longus muscle.

Baseline to peak amplitude, which reflects the muscle mass, is generally larger for the quadriceps muscle than for the extensor digitorum longus muscle.

The number of fluctuations is generally higher for the quadriceps muscle than the extensor digitorum longus muscle. This indicates that the quadriceps muscle, which is more involved in the PA, also takes care of coordination and imbalance problems during it.

The duration of the squats is quite consistent across the two muscles. This consistency suggests that both muscles follow a similar timing pattern during the squat. However, the duration of squats varies between the participants. The variation in duration indicates that the participants’ ability to make directional changes vary.

### 3.2 Characteristics of the EBI signals obtained during lunges

**FIGURE 7:**
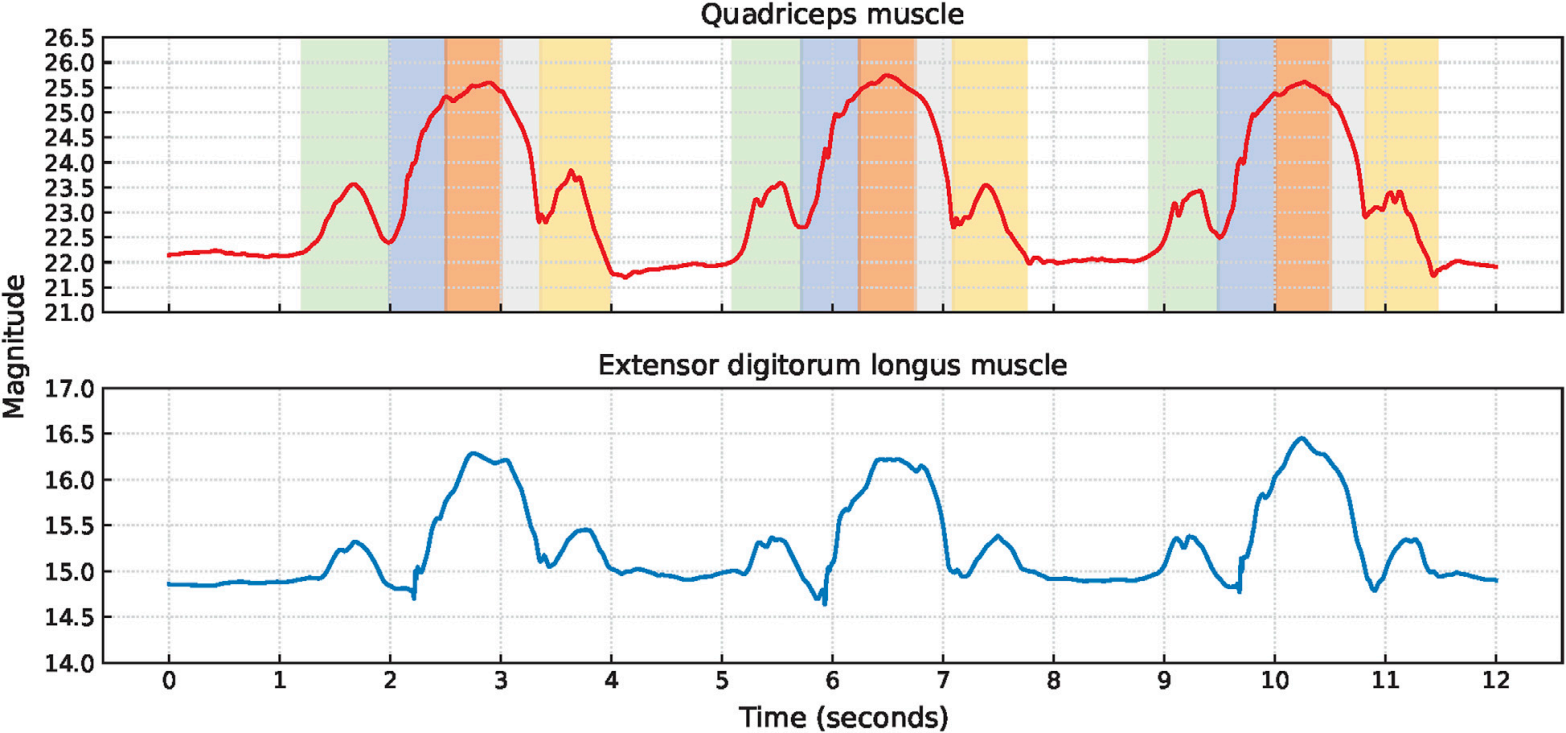
The signal characteristics of the EBI signal for P6 performing lunges. The approximate timings for the key phases are color-coded: green for the step forward with the sensor leg, blue for bending knees, orange for lunge position, gray for leg extension, and yellow for taking the leg back in the EBI signal obtained from the quadriceps muscle. The V-shapes represent the foot of the sensor leg making contact with and leaving the ground, respectively.

Lunge cycles were extracted from the quadriceps muscle for all 11 participants and from the extensor digitorum longus muscle for 8 participants. The signal characteristics of the EBI signals obtained from the quadriceps muscle and the extensor digitorum longus muscle of P6 are presented in Figure 7. In each lunge cycle, the first small hill represents the step forward while the first V-shape indicates when the foot touches the ground. The top of the larger hill represents the lunge position, where the knees are bent 90°. The second V-shape represents when the foot leaves the ground on the way back to the starting position, and the second small hill represents taking the leg back.

Although similar signal characteristics were observed for both muscles, the amplitude of the EBI signal obtained from the quadriceps muscle was significantly higher than that from the extensor digitorum longus muscle.

#### 3.2.1 Variations in the EBI characteristics

Several variations in the EBI signals obtained during the lunges were observed, including plateaus, small hills, unclear second small hills, and fluctuations.

**FIGURE 8:**
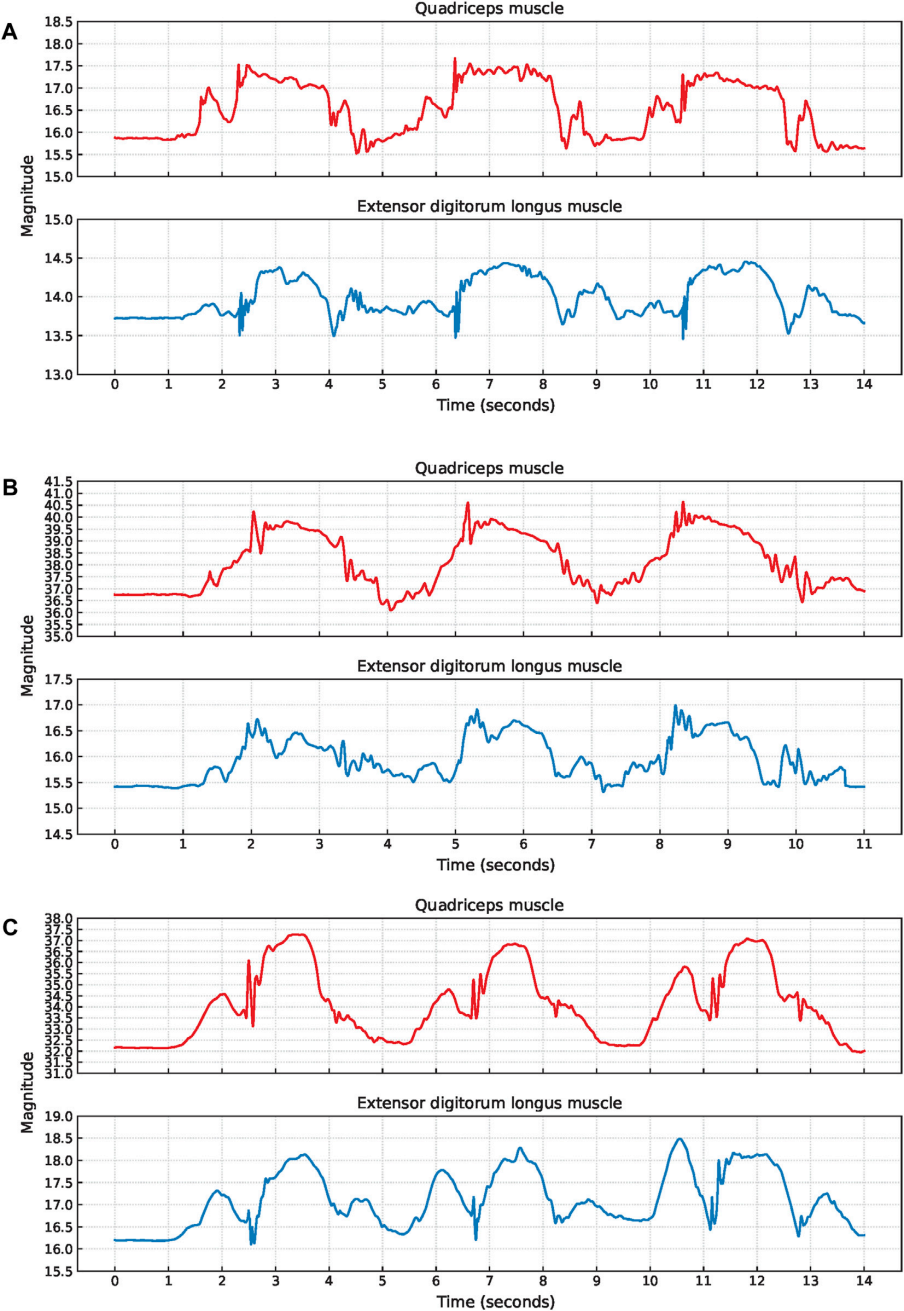
Examples of EBI signal characteristics during lunges where **(A)** shows a plateau at the bigger hill (P3), **(B)** shows unclear V-shapes (P11), and **(C)** shows an unclear second small hill (P9).

Two participants exhibited a clear plateau in the EBI signal obtained from the quadriceps muscle while holding the lunge position. Video analysis revealed that both participants paused in this position before returning to the starting position. One participant (P3) displayed a fluctuating EBI signal with a plateau, as shown in Figure 8A. The cause of this fluctuation could not be determined from the video observations.

The signal characteristics of the small hills varied, particularly concerning the presence of the V-shape. Four participants had an unclear V-shape, and an example of this (P11) is shown in Figure 8B. Video analysis indicated that two of these four participants put their toes down first when stepping forward, and that one took a short step forward.

Additionally, three participants exhibited an unclear second small hill, and an example of this (P9) is shown in Figure 8C. Among these three participants, one took a short step forward, while the other two appeared to stand up before returning the leg to the starting position.

Examples of fluctuations in the EBI signal are shown in Figures 8A–C.

#### 3.2.2 Calculated features

**FIGURE 9:**
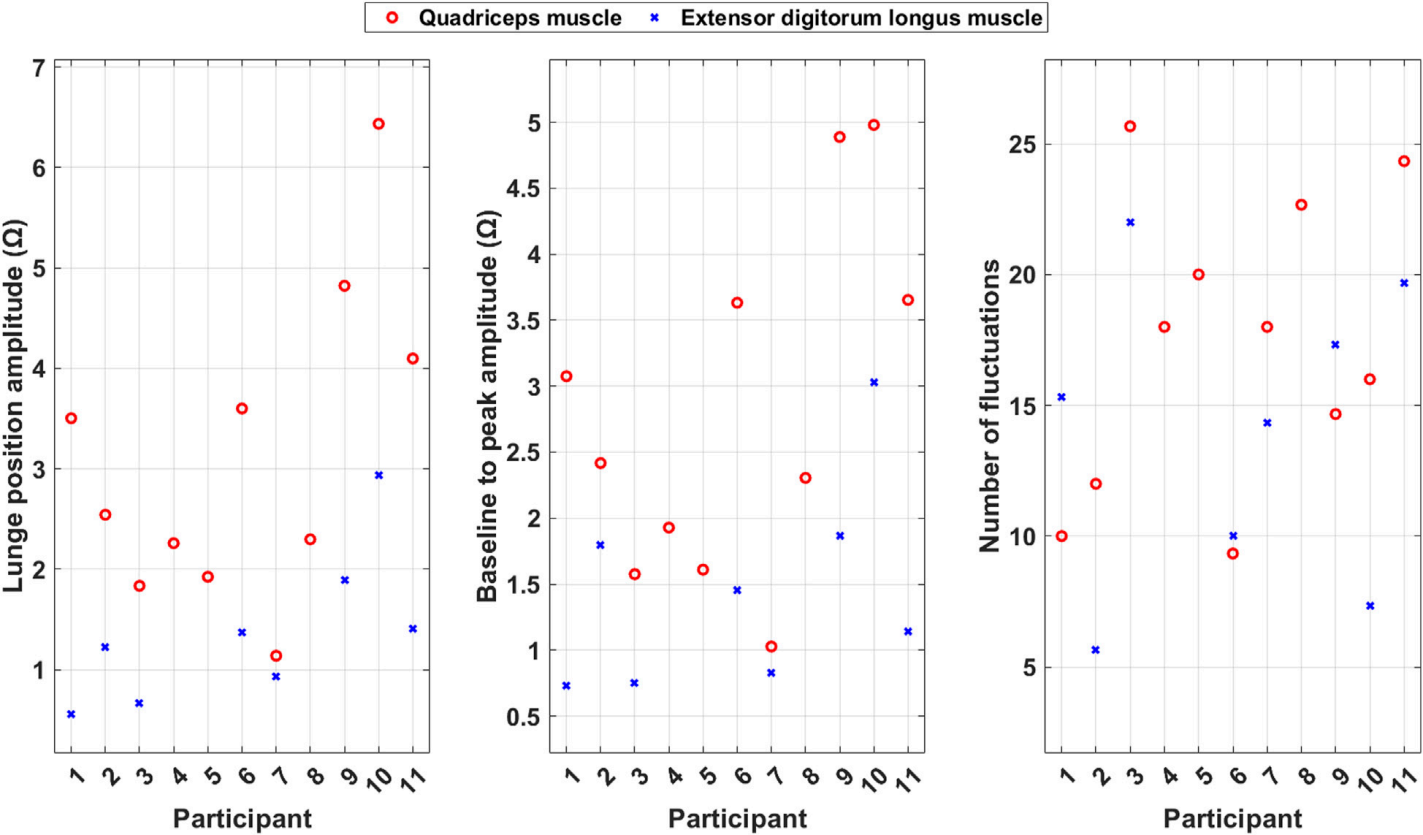
Scatter plots of the average calculated features during lunges for each included participant.

Figure 9 shows scatter plots of the average calculated features for each included participant and indicates variability in functionality between the quadriceps muscle and extensor digitorum longus muscle.

The lunge position amplitude is generally larger for the quadriceps muscle than for the extensor digitorum longus muscle. This indicates that the quadriceps muscle has a stronger and more consistent contraction during the PA than the extensor digitorum longus muscle.

Baseline to peak amplitude, which reflects the muscle mass, is generally larger for the quadriceps muscle than for the extensor digitorum longus muscle.

The number of fluctuations is higher for the quadriceps muscle than for the extensor digitorum longus muscle for some of the participants, but for others, the extensor digitorum longus muscle has more fluctuations. This indicates that the participants use the muscles in different ways to take care of coordination and imbalance problems during the PA.

### 3.3 Characteristics of the EBI signals obtained during balance walk

The analysis of balance walk included EBI signals obtained from the quadriceps muscle for 9 participants and from the extensor digitorum longus muscle for 8 participants.

**FIGURE 10:**
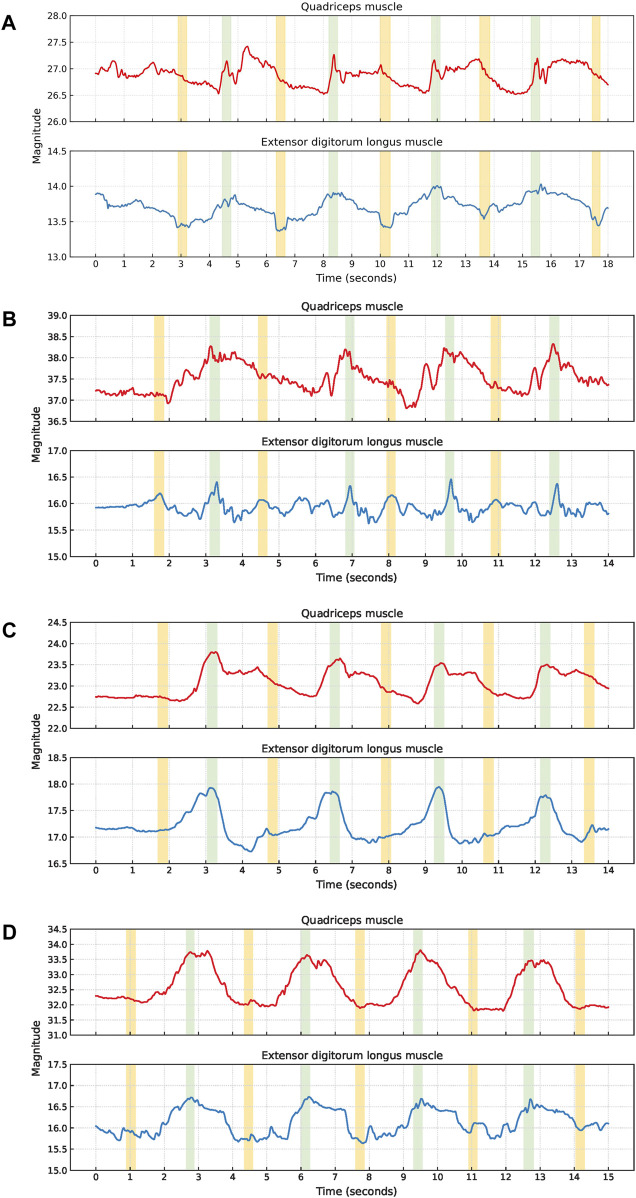
Examples of EBI signal characteristics during balance walk. The approximate timings for the key phases are color-coded: green for the swing phase of the sensor leg, and yellow for the swing phase of the non-sensor leg. A small hill is observed during the swing phase of the sensor leg in **(A–D)**. During the swing-phase of the non-sensor leg, **(A)** U-shapes are observed for P8’s extensor digitorum longus muscle, **(B)** shows small hills for P11, **(C, D)** no U-shape or small hill are observed for P2 and P9. The EBI signal characteristics vary between steps and participants.

As shown in Figure 10, the signal characteristics of the EBI signals vary significantly. Due to unclear signal characteristics and the fact that phases were sometimes impossible to detect without video observations, no balance walk cycles were extracted. The common signal characteristics observed in the EBI signals are that the magnitude increases when a participant begins to move the sensor leg, and that the magnitude is highest during the swing phase of the sensor leg. The magnitude then decreases and remains low during the swing phase of the non-sensor leg. A small hill often occurs during the swing phase of the sensor leg. Four examples of EBI signals exhibiting the small hill during the swing phase of the sensor leg are provided in Figure 10.

The swing phase of the non-sensor leg is only occasionally visible. It can occur as a U-shape or a small hill. The U-shape is observed in the EBI signal obtained from the extensor digitorum longus muscle of P8 in Figure 10A. Small hills are observed for P11 in Figure 10B. For P2 and P9, no U-shapes or small hills are observed during the swing phase of the non-sensor leg in Figures 10C, D.

The most significant variation in the EBI signal characteristics occurs in between the swing phases, i.e., when both feet are on the ground. This variation reflects the different roles of the muscles in shifting the center of gravity and maintaining balance. As shown in Figure 10, the EBI signal between the swing phases varies between participants, but also between the steps for each participant.

The approximate amplitudes varied among the participants. For the quadriceps muscle it ranged from 0.89 to 3.7 Ω, with a mean of 1.78 Ω, while it ranged from 0.46 to 1.57 Ω, with a mean of 1.03 Ω for the extensor digitorum longus muscle.

**FIGURE 11:**
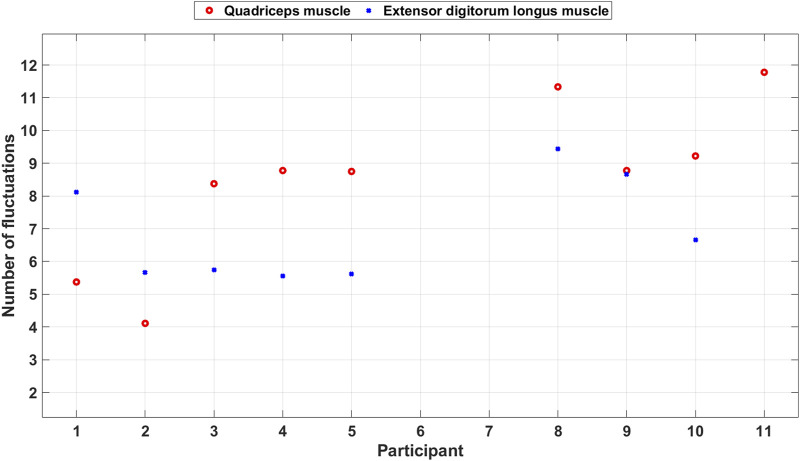
Scatter plot of the number of fluctuations during balance walk for each included participant.

Figure 11 shows a scatter plot of the average number of fluctuations per step among the included participants. The number of fluctuations is often higher for the quadriceps muscle than for the extensor digitorum longus muscle except for two participants. This indicates that the participants use the muscles in different ways to take care of coordination and imbalance problems during the PA.

The EBI signal from the extensor digitorum longus muscle of P11 did not display the typical characteristics observed in other participants’ EBI signals and was thereby excluded. Looking at that EBI signal we can see a lot of fluctuations. Also, for the quadriceps muscle P11 had a lot of fluctuations. Video observations show that P11 struggled with the balance and wobbled during the balance walk.

### 3.4 Characteristics of the EBI signals obtained during short jump

Short jump cycles were extracted from the quadriceps muscle for 7 participants and from the extensor digitorum longus muscle for 9 participants.

**FIGURE 12:**
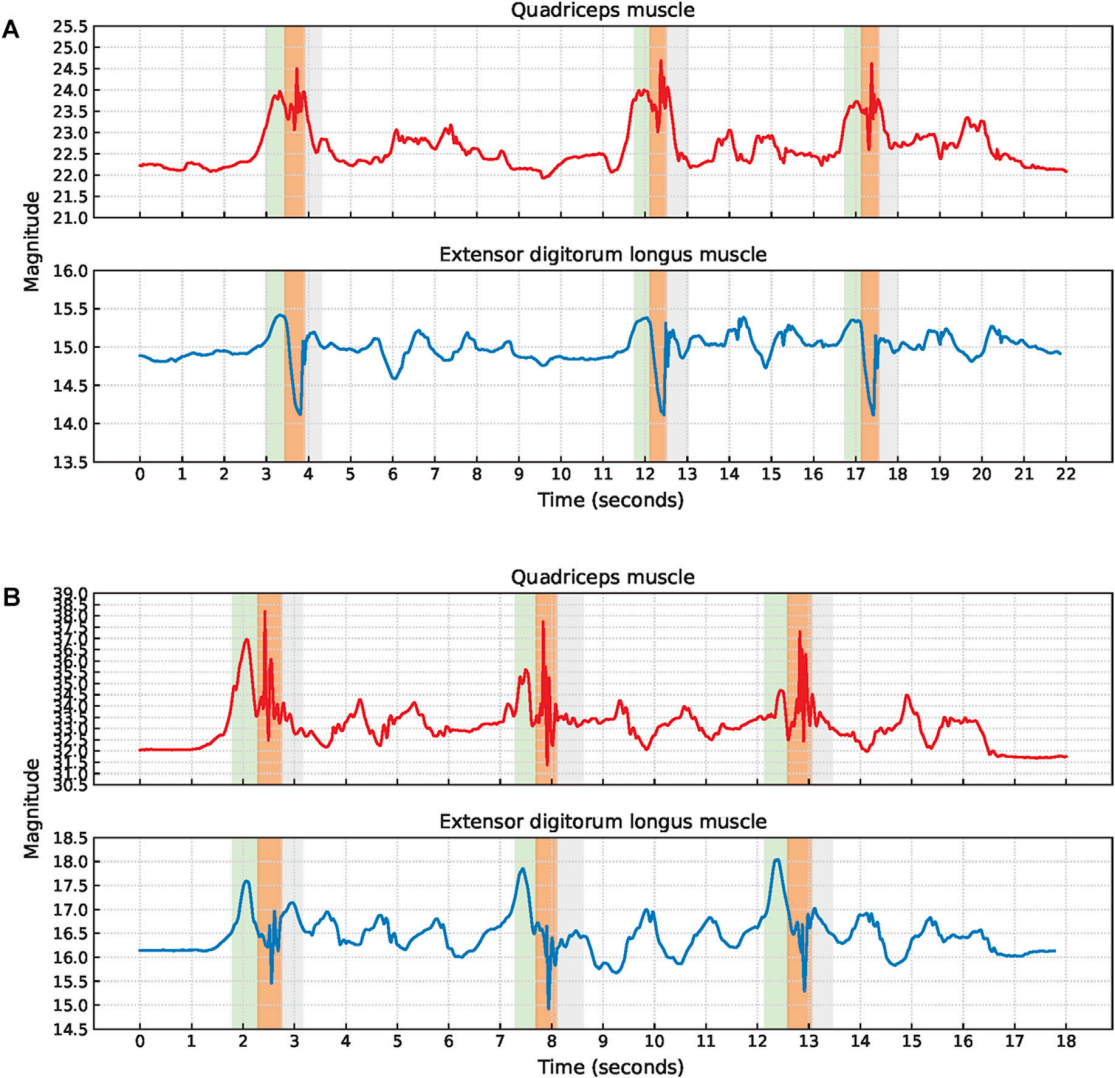
Examples of EBI signal characteristics during short jumps where **(A)** a V-shape during the landing is observed for the extensor digitorum longus muscle while the signal fluctuates for the quadriceps muscle (P6), and **(B)** the signals are fluctuating during the landing for both muscles (P9). The approximate timings for the key phases are color-coded: green for step forward with the sensor leg, orange for landing with the sensor leg, and gray for landing with the non-sensor leg.

The signal characteristics of the EBI signals obtained during short jumps were often unclear and the phases were sometimes impossible to detect without video observations. Therefore, no short jump cycles were extracted. Common signal characteristics observed are a small hill when stepping forward with the sensor leg, a V-shape or fluctuations while landing with the sensor leg, and another small hill when landing with the non-sensor leg, Figure 12.

The V-shape was observed for seven of the EBI signals obtained from the extensor digitorum longus muscle. The other two EBI signals exhibited fluctuations where the V-shape typically appeared.

For two participants, the V-shape was also present in the EBI signal obtained from the quadriceps muscle. The EBI signal for the remaining five included participants exhibited fluctuations instead of the expected V-shape.

P6 landed with bent legs after each short jump. In Figure 12A, this is reflected by an increased EBI signal magnitude for the quadriceps muscle which decreased when P6 straightened the legs.

There were also differences in the approximate amplitude among the participants. For the quadriceps muscle, the amplitudes ranged from 1.68 to 6.81 Ω, with a mean of 3.80 Ω, and for the extensor digitorum longus muscle exhibited a range of 0.92–3.12 Ω, with a mean of 1.65 Ω.

## 4 Discussion

While three-dimensional Optical Motion Capture systems or IMU sensors provide information on the direction of body movement, they cannot be used to analyze the muscles involved. In addition, IMU sensors’ capability to detect age related deteriorations in how to perform a PA is low (Swanson and Fling, 2021). By using EBI technology for human motion recognition, it might be possible to get information on how the muscles handle PAs performed.

In this study, we investigated the potential of using the EBI technique for human motion recognition for being able to analyze more than the change in body directions that occur while performing different PAs. By analyzing EBI signals obtained from the quadriceps muscle and extensor digitorum longus muscle while performing four lower body PAs (squats, lunges, balance walk, short jumps), we found that the signal characteristics of the EBI signals differ between the four PAs.

The sensor placements were selected to target muscles involved in the PAs. However, the study does not evaluate the optimal sensor positions for these kinds of measurements. Interestingly, the signal characteristics were similar for both muscles.

The selected PAs were chosen due to their relevance in assessing functional movements and because they engage multiple lower body muscles. They also require varying levels of strength, power, balance, speed, and coordination. An objective of the PRE-fall project is to find sensor solutions capable of detecting differences in sensor signals obtained from working muscles between different age groups. This study represents a first step towards that goal by characterizing EBI signals for the four selected PAs for young, healthy individuals in the age group 20–30 years old.

Variations in the EBI signal characteristics across participants suggest individual differences in muscle activation patterns. This could be due to factors such as fitness level, muscle mass, or how a participant executes the PA. However, the exact reasons for these variations were not always evident from the visible movements captured in the videos. This indicates that underlying physiological or biomechanical factors beyond what can be visually observed might be at play.

The complexity of a PA directly influences the variation in how participants perform the PA and engage different muscles. Squats, a relatively simple PA primarily involving the bending of legs and standing up, showed typical EBI characteristics for all participants except for participant P5, whose quadriceps muscle readings differed. Since squats do not significantly challenge balance, variations in how participants engaged the quadriceps muscle and extensor digitorum longus muscle were minimal. In contrast, lunges and short jumps, despite clear instructions, allowed for some variability in execution and different muscle activation patterns. The balance walk, while limited in execution variability, required different muscle engagement strategies to maintain stability.

Variations in balance walk were often observed when the participant shifted the gravidity from one leg to another while having both feet on the ground. For short jumps, it was difficult to distinguish between the short jump and the walking backward to the starting position. This may be due to the relatively short, 70 cm, jump which can resemble a large step. Thereby, it is difficult to differentiate between them in the EBI signal. A more forceful execution of the short jump would likely make it easier to detect.

In general, the quadriceps muscle exhibits higher magnitudes and greater variability across most features compared to the extensor digitorum longus muscle. This is not surprising given the quadriceps muscles’ larger muscle volume which aligns with its role as the primary muscle engaged during squats and lunges. The extensor digitorum longus muscle, on the other hand, functions primarily to stabilize the leg and foot during these movements but does not significantly contribute to the core of action. The extensor digitorum longus muscle contributes more to the core of action during balance walk and short jumps. However, given its’ relatively smaller muscle volume, the approximate amplitudes remain small.

Lunges and balance walk resulted in more fluctuations in the EBI signal than squats. For one participant, the balance walk led to pronounced fluctuations, with the video showing visible balance issues and wobbling. Thereby, at least some of the fluctuations seem to relate to muscles trying to compensate for the imbalance. Additionally, the movement during these PAs may have impacted on the contact between the skin and the electrodes, potentially influencing the EBI signal. However, if that was the case, this would likely occur during fast movements, such as squats, rather than slow activities like balance walk.

Since each participant performed each PA only once, we cannot determine whether the observed fluctuations, regardless of their causes, would have occurred consistently across repeated sessions. Similarly, we cannot confirm if the variations in the EBI characterization would be present in every repeated session.

None of the participants were excluded from more than two PAs. Three participants’ EBI signals were excluded from two PAs, with exclusion occurring for only one muscle per PA for each of them. Additionally, three participants’ EBI signals were excluded from one PA for both muscles. The excluded EBI signals exhibited characteristics unrelated to the PA, including both small and large fluctuations in magnitude that were not attributable to the participants’ movements. These anomalies may have resulted from baseline artifacts, potentially caused by the cables or electrode skin contact. To reduce such interference in future studies, it is recommended that the cables are more securely fixed than in this study.

An integrated method of video analysis and waveform observation allowed for a comprehensive understanding of the PA cycles and their EBI signal characteristics. Despite having access to videos, a large part of the EBI signals would have been impossible to understand. However, the proposed cycle extraction algorithm for squats and lunges requires the calculation of baseline, mean, peak, and thresholding data for each participant. This process is time-consuming and demands a significant number of human resources since all signals and videos need to be carefully checked. There is a need for a more automated approach to reduce human intervention.

The variations in EBI signal characteristics provide valuable insights into the different muscle activity patterns during PAs. In sport and rehabilitation, there is an opportunity to use EBI signals to see how the person executes a specific PA and thereby help the person to execute the PA optimally.

Future work includes further investigation with more participants to determine whether these EBI signal characteristics vary for people in other age groups and across other PAs. Additionally, data from other types of sensors needs to be collected and analyzed in order to better understand differences between people while performing the PAs.

## 5 Conclusion

This study highlights the potential of EBI as a novel technique for analyzing and characterizing lower body muscle activity during PAs such as squats, lunges, balance walk, and short jumps. The findings show unique EBI signal characteristics for each PA and reveal insights into muscle activation patterns. However, the EBI signal characteristics differ between participants and each cycle of a PA for an individual participant. The result highlights the influence of PAs complexity and shows larger amplitudes for larger muscle mass and for the more engaged muscle.

The characteristics of EBI signals are promising for analyzing lower body PAs. Each evaluated PA exhibited unique EBI signal characteristics. The variability in how PAs are executed leads to variations in the EBI signal characteristics, which, in turn, can provide insights into individual differences in how a person executes a specific PA.

## Data Availability

The raw sensor data supporting the conclusions of this article will be made available by the authors upon request, without undue reservation.

## Footnotes

1 Analog Devices. “MAX30009EVKIT”; https://www.analog.com/en/resources/evaluation-hardware-and-software/evaluation-boards-kits/max30009evkit.html#eb-overview [Accessed February 1, 2023].

2 Analog Devices. “MAX86178EVKIT”; https://www.analog.com/en/resources/evaluation-hardware-and-software/evaluation-boards-kits/max86178evkit.html#eb-overview [Accessed July 1, 2023].

3 Mälardalen University. “PRE-fall project”; https://www.mdu.se/en/malardalen-university/research/research-projects/pre-fall [Accessed October 2, 2024].

4
https://matplotlib.org/

5
https://scipy.org/
